# Negative pressure wound therapy with intermittent irrigation for treatment of post-traumatic giant abscess: A case report

**DOI:** 10.1016/j.ijscr.2022.107068

**Published:** 2022-04-11

**Authors:** Mohammad Abidali, Frank Bauer, Marc Gottlieb, Ali Abidali

**Affiliations:** a American University of the Caribbean School of Medicine, 1 University Drive at Jordan Dr, Cupecoy, Sint Maarten (Dutch Part); b HonorHealth Surgical and Trauma Specialist, 7351 E. Osborn Rd #200B, Scottsdale, AZ 85251, United States of America; c University of Cincinnati College of Medicine, 231 Albert Sabin Way, Cincinnati, OH 45267, United States of America

**Keywords:** Negative pressure wound therapy (NPWT), Hepatic abscess, Wound vac, Wound healing, Instillation, Bile leak

## Abstract

**Introduction:**

Negative pressure wound therapy (NPWT), also called vacuum-assisted closure, is an adjunctive therapy used to manage open wounds that apply subatmospheric pressure to the wound surface. The therapeutic effects of NPWT are exerted by stabilizing the wound environment, increasing blood flow, and macro-deformation of wounds that initiate granulation tissue formation.

**Case presentation:**

We present a case of a 28-year-old Caucasian male who developed a giant non-resolving hepatic abscess secondary to a gunshot wound (GSW) to the upper right abdomen. The abscess was successfully treated with open debridement followed by NWPT with instillation therapy. Significant reduction in abscess diameter and cessation of trauma-induced bile leak was observed following 15 days of wound vac treatment.

**Discussion:**

Wound vac treatment was essential in this patient due to the inherent erosive properties of bile that damage surrounding tissue and perpetuate opportunistic growth of pathogenic microbes. Prior to standard NPWT treatment, debridement of devitalized tissues and infection should be managed; however, instillation therapy has permitted NPWT to be used in the presence of infection or as an adjuvant to surgical infection management.

**Conclusion:**

NPWT is indicated for a wide range of acute and chronic wounds; however, the utilization of NPWT to treat abscesses remains unclear.

## Highlights

• This case exhibits the novel use of NPWT to treat a hepatic abscess with bile leak.
• Instill wound VAC can reduce the bacterial wound burden and enhance wound healing.
• Hepatic abscess after trauma is a life-threatening condition with high morbidity.
• Operative management may be necessary for large non-resolving hepatic abscesses.

## 1 Introduction

Negative pressure wound therapy (NPWT) is a non-invasive technique that has been broadly utilized in surgical practices for acute and chronic open wound management [1]. However, limited literature is available for the usage of NPWT on open abdominal (OA) management of liver abscesses with associated bile leak [2], [3]. Small liver abscesses are often treated with antibiotics and are self-resolving without surgical intervention, but larger abscesses may require percutaneous computer-tomography (CT) guided drains [4]. Abscesses that will not respond to conservative management may require additional surgical intervention. Marsupialization can be an effective technique to manage a portion of these deep organ space abscesses of the liver. In these cases of marsupialization, creating a direct window to the wound, NPWT can aid in OA wound healing by increasing blood flow, decreasing wound healing time, and reducing pain associated with dressing changes [5]. Negative pressure wound therapy with instillation and dwell time (NPWTi-d) allows volumetrically controlled fluid instillation and irrigation, aiding in removing intrabdominal cellular debris and fluids and reducing the bacterial wound burden—resulting in shorter hospital stays [5]. The work has been reported in line with the SCARE criteria [6].

## 2 Case presentation

A 28-year-old male was brought to an outside hospital with a gunshot wound (GSW) to the upper right abdomen. Exploratory laparotomy revealed penetrating liver trauma that resulted in massive liver hemorrhage and bile leak. Damage control laparotomy was performed for stabilization and multiple peri-hepatic drains were placed—a bile leak developed within 24 h following the initial trauma. After achieving physiologic stability, the abdomen was closed with multiple peri-hepatic drains left in situ for the ongoing control of bile leak. Subsequently, an endoscopic retrograde cholangiopancreatography (ERCP) was performed to place an internal stent to promote bile drainage. The patient was discharged from his initial hospital with drains remaining in place.

**Fig. 1:**
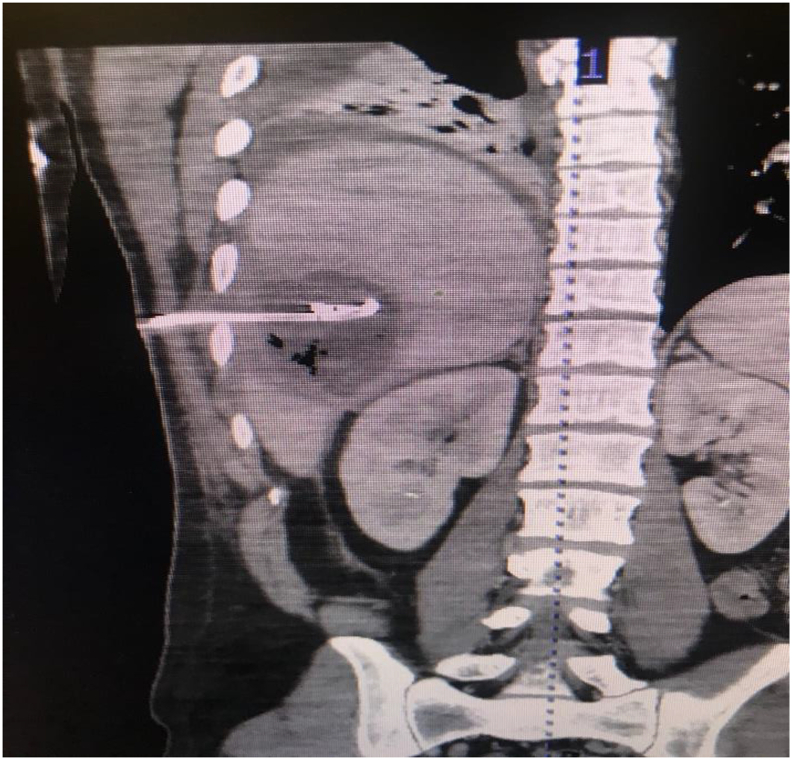
Coronal CT showing hepatic abscess with drains.

**Fig. 2:**
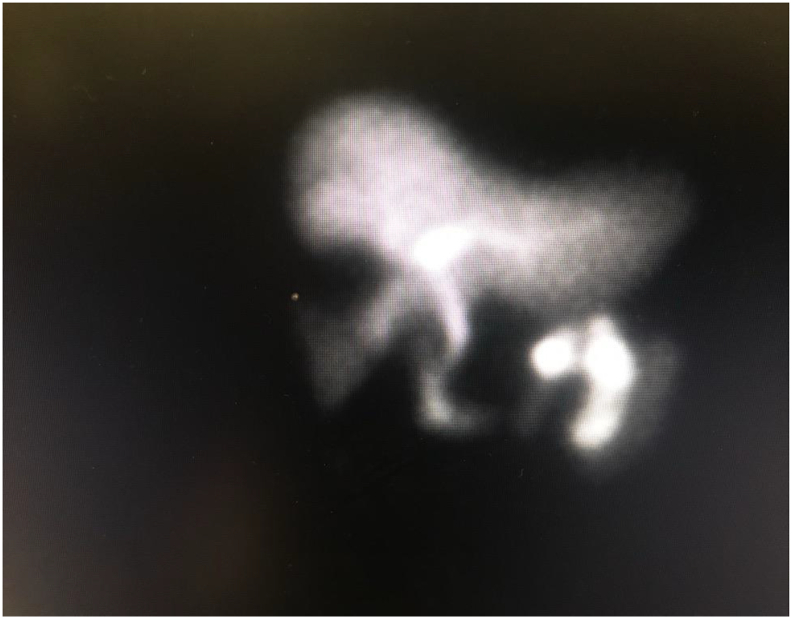
HIDA scan without evidence of bile leak.

**Fig. 3:**
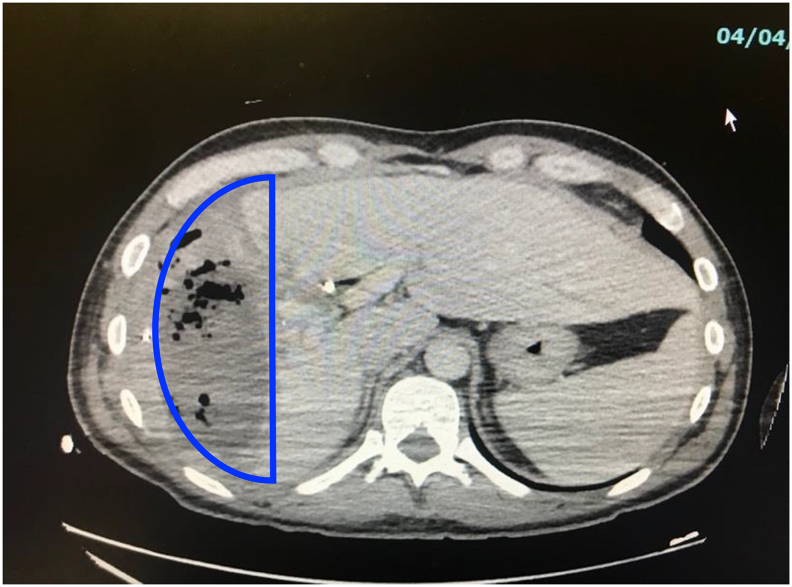
CT scan showing giant hepatic abscess with occult bile leak. The outlined arch indicates the liver nestled within the ribs, preventing natural wound contractile forces.

**Fig. 4:**
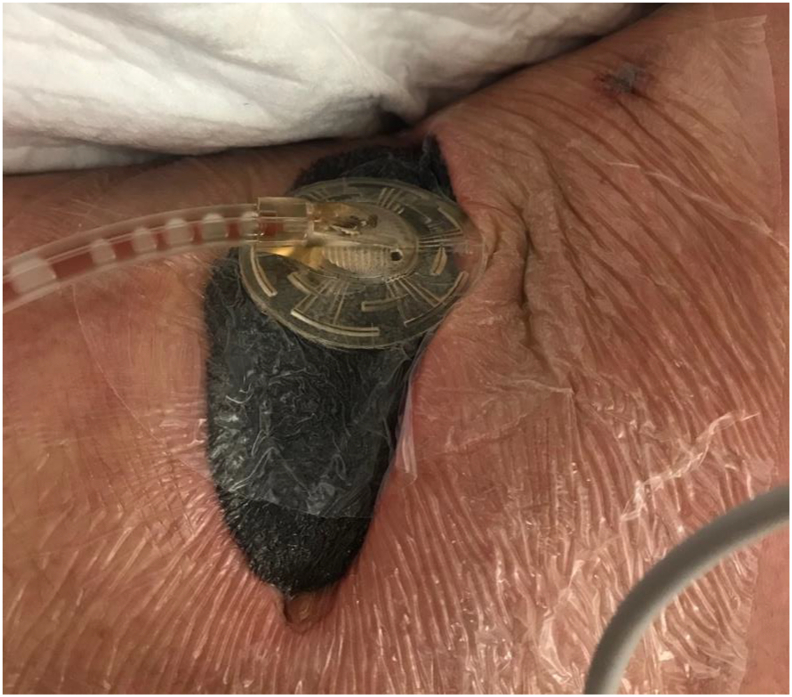
Patient with wound vac.

**Fig. 5:**
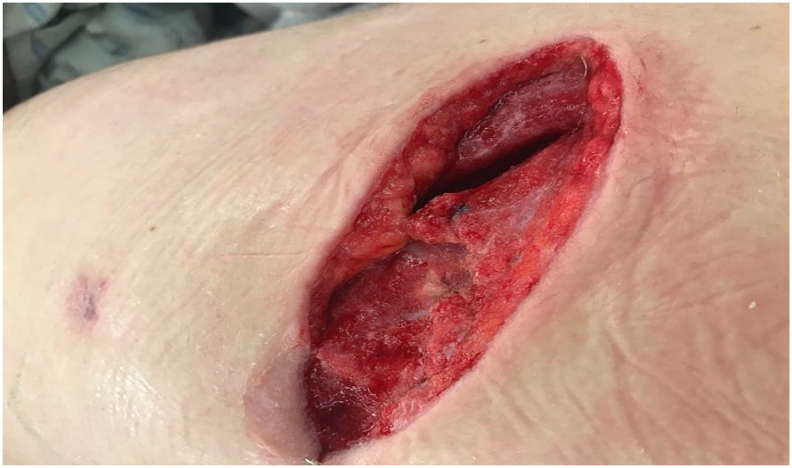
The patients wound after NPWT. Ready for closure by secondary intention.

**Fig. 6:**
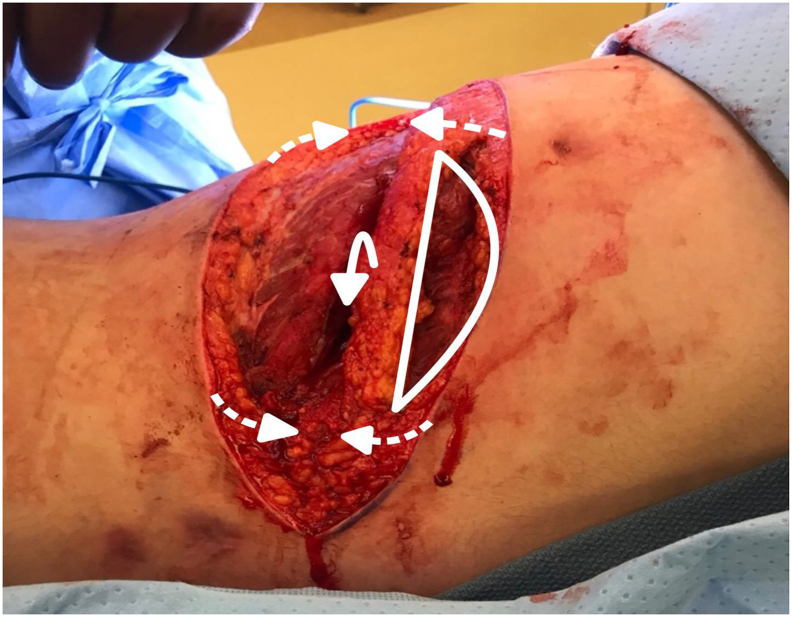
Subcutaneous fat was grafted (indicated by the arc) and utilized to close the dead space in the intercostal area (solid arrow). Skin flaps were then employed to facilitate complete wound closure by secondary intention.

The patient arrived septic at our Emergency Department with acute postoperative pain and fever. Cultures of the drain revealed *Pseudomonas aeruginosa*, although blood cultures were negative. Computerized tomography (CT) revealed a 15.1 × 6.8 × 14.3 cm liver abscess that had developed in the previous wound bed. Our initial decision was to treat non-operatively via antibiotics and place an additional CT-guided drain via Interventional Radiology [Fig. 1]. A hepatobiliary iminodiacetic acid (HIDA) scan showed normal hepatobiliary ducts without obstruction or leakage [Fig. 2]. CT imaging demonstrated no response to a week of conservative management and the patient had increasing systemic inflammatory response syndrome (SIRS) with frailty and malnutrition. Thus, the abscess was operatively debrided by an open incision via trans-diaphragmatic intercostal approach, similar to a Clagett but without costal resection. The wound was then managed with serial wound vac changes in the operating room utilizing a wound vac with a white sponge. Once purulence and necrotic tissue were decreased, we discovered an occult bile leak into the wound bed. [Fig. 3]. A NPWTi-d wound vac was placed that irrigated saline for 5 min/h to aid in the removal of bile and promote wound healing [Fig. 4]. CT showed a 7 × 2 × 2 cm liver abscess 10 days status-post wound vac treatment. The patient remained on wound vac therapy for 15 days, resolving the hepatic abscess and bile leak. The wound was ultimately closed by secondary intention [Fig. 5] with simple subcutaneous flaps to fill the dead space and re-approximate the wound edges [Fig. 6]. In this case, operative management in conjunction with i-NPWT wound vac was used to successfully treat a giant pyogenic hepatic abscess that had failed optimum conservative management with drainage and intravenous antibiotics.

## 3 Discussion

Selective non-operative management (NOM) has increased in popularity among surgeons for hepatic abscesses. NOM of hemodynamically stable patients inflicted with penetrating liver wounds had a significantly lower mortality rate than the operative group with success rates greater than 85% [7], [8]. Surgically managed hepatic abscesses have drastically decreased; however, certain cases may still have a role.

Negative pressure wound therapy with instillation and dwell time (NPWTi-d) has been shown to promote wound healing by increasing granulation tissue formation, removing cellular debris and devitalized tissue, and irrigating caustic fluids [5]. A retrospective study compared NPWTi-d for extremity or trunk wounds to a control group who received NPWT without instillation showed that patients who received NPWTi-d required fewer surgical debridements, experienced a shorter average length of stay, a reduced length of therapy and time of wound closure [5].

Pyogenic hepatic abscesses are life-threatening conditions often arising from intra-abdominal infection or trauma. Small hepatic abscesses are often self-resolving with antibiotics alone, while larger hepatic abscesses often require percutaneous drains. Surgery is often considered for non-resolving hepatic abscesses when percutaneous drains are unsuccessful [4]. There are certain cases where marsupialization of the deep space infection of the liver can be advantageous. In this case, it allowed for the use of NPWT with instillation therapy. NOM has shown great promise in managing small liver abscesses; however, limited literature is available to utilize NPWT for managing large non-resolving hepatic abscesses.

Various aspects may explain why this hepatic abscess did not resolve with drains and antibiotics alone. Large abscesses are more susceptible to failure with conservative therapy. There is likely poor antibiotic penetration deep within the abscess cavity. Certain bacteria are more difficult to resolve with conservative management; in this case, pseudomonas was present. Wounds heal by contracture, but contracture was not possible since the liver was nestled within the arches of the ribs [Fig. 3]. These arches are very stable structures that prevent the natural contractile forces of wound healing. Finally, continuous occult bile leak promoted ongoing damage to the surrounding tissue.

Wounds infected with biofilm-forming bacteria have posed additional difficulties in wound healing for decades. Free leakage of bile into the peritoneum perpetuates the growth of opportunistic biofilm-forming *Pseudomonas aeruginosa*, allowing the secretion of its virulence factors, pseudolysin and protease IV, which affects the survival rate of fibroblast, impede cell migration, and inhibit angiogenesis [9]. NPWT is an effective strategy for managing contaminated wounds by reducing virulence factors and bacteria proliferation [10].

Trauma-induced bile leak into the peritoneal cavity increases the incidence of inflammation, infection, and healing time. Bile acids and salts reduced fibroblast viability in a time and dose-dependent manner, reducing healing time [10], [11], [12]. NPWTi-d provides a modality for introducing fluids that irrigate bile and other unwanted cellular debris that impede wound healing [1]. Once the wound cavity was effectively debrided, antibiotics were notably more effective.

This patient's trauma and sepsis led to a hypermetabolic state with a steadily declining BMI, raising malnutrition suspicion. Total parenteral nutrition (TPN) was initiated to restore nitrogen balance and promote an anabolic state. Wound healing increases energy needs for sufficient collagen synthesis, immune function, and wound tensile strength [13]. Failure to satisfy metabolic demands in trauma and septic patients may lead to suboptimal nutrition that prolongs wound healing. Endogenous cytokine release from activated leukocytes utilizes proteins to sustain normoglycemia [14]. Protein deficiency slows down fibroblast and collagen development—increasing the risk of wound dehiscence and reducing the strength of scar tissue [15]. These patients require nutritional supplementation to counter a negative nitrogen balance.

## 4 Conclusion

Hepatic abscesses arising from penetrating trauma are known sequelae that can be complex and challenging to treat. The presented case demonstrates the successful novel use of NPWTi-d to treat a giant hepatic abscess with associated bile leak.

## Sources of funding

All authors declare that no funding was received for this research.

## Consent

Written informed consent was obtained from the patient for publication of this case report and accompanying images. A copy of the written consent is available for review by the Editor-in-Chief of this journal on request.

## Ethical approval

This study is exempt from ethical approval.

## Author contribution

Mohammad Abidali, Frank Bauer and Marc Gottlieb, Ali Abidali were all major contributors in writing, editing, and revising the manuscript. Frank Bauer and Marc Gottlieb were also directly involved in the clinical case.

## Registration of research studies

N/A.

## Guarantor

Mohammad Abidali.

## Provenance and peer review

Not commissioned, externally peer-reviewed.

## Declaration of competing interest

The authors have no conflicts of interest to disclose.
